# The critical incident inventory: characteristics of incidents which affect emergency medical technicians and paramedics

**DOI:** 10.1186/1471-227X-12-10

**Published:** 2012-08-03

**Authors:** Janice Halpern, Robert G Maunder, Brian Schwartz, Maria Gurevich

**Affiliations:** 1 Department of Psychiatry, Mt. Sinai Hospital and University of Toronto, 244 Dupont Street, Toronto, ON M5R 1V7, Canada; 2 Department of Psychiatry, Mt. Sinai Hospital and University of Toronto, 600 University Avenue, Toronto, ON M5G 1X5, Canada; 3 Public Health Ontario, and Department of Family and Community Medicine, University of Toronto, 1075 Bay St., 11th floor, Toronto, ON Canada; 4 Previous Affiliation: Sunnybrook Health Sciences Centre, University of Toronto, Toronto, ON Canada; 5 Department of Psychology, Ryerson University, 350 Victoria Street, Toronto, ON M5B 2K3, Canada

## Abstract

**Background:**

Emergency medical technicians (EMTs) and paramedics experience critical incidents which evoke distress and impaired functioning but it is unknown which aspects of incidents contribute to their impact. We sought to determine these specific characteristics by developing an inventory of critical incident characteristics and testing their relationship to protracted recovery from acute stress, and subsequent emotional symptoms.

**Methods:**

EMT/paramedics (n = 223) completed a retrospective survey of reactions to an index critical incident, and current depressive, posttraumatic and burnout symptoms. Thirty-six potential event characteristics were evaluated; 22 were associated with peritraumatic distress and were retained. We assigned inventory items to one of three domains: situational, systemic or personal characteristics. We tested the relationships between (a) endorsing any domain item and (b) outcomes of the critical incident (peritraumatic dissociation, recovery from components of the Acute Stress Reaction and depressive, posttraumatic, and burnout symptoms). Analyses were repeated for the number of items endorsed.

**Results:**

Personal and situational characteristics were most frequently endorsed. The personal domain had the strongest associations, particularly with peritraumatic dissociation, prolonged distressing feelings, and current posttraumatic symptoms. The situational domain was associated with peritraumatic dissociation, prolonged social withdrawal, and current posttraumatic symptoms. The systemic domain was associated with peritraumatic dissociation and prolonged irritability. Endorsing multiple characteristics was related to peritraumatic, acute stress, and current posttraumatic symptoms. Relationships with outcome variables were as strong for a 14-item inventory (situational and personal characteristics only) as the 22-item inventory.

**Conclusions:**

Emotional sequelae are associated most strongly with EMT/paramedics’ personal experience, and least with systemic characteristics. A14-item inventory identifies critical incident characteristics associated with emotional sequelae. This may be helpful in tailoring recovery support to individual provider needs.

## Background

Emergency medical technicians and paramedics (EMT/paramedics) are subject to critical incidents, defined as stressful workplace incidents that evoke acute distress and which may impair functioning in the short- or long-term [1]. Researchers have compiled lists of the qualities of critical incidents based on EMT/paramedics’ reports, which include characteristics of the patient (e.g. a child, someone related to the ambulance crew, someone who sustained gruesome injuries or died), the situation (e.g. danger to ambulance personnel, problems with how the call was relayed), and the EMT/paramedic’s personal response (e.g. feeling helpless) [2-5]. These lists suggest that EMT/paramedics may be able to identify the types of situations that cause acute distress. However, the tantalizing question remains: which acutely stressful incidents result in ongoing symptoms and impaired functioning?

Work stress, including the effects of critical incidents, burdens EMT/paramedics and their organizations, and may interfere with patient care. Posttraumatic Stress Disorder (PTSD) is often associated with EMT/paramedics’ critical incidents and is found in 12% to 20% of EMT/paramedics compared to a community prevalence of 1-3% [6]. Burnout, depression and anxiety have also been attributed to critical incidents [5]. These syndromes likely contribute to EMT/paramedics’ high sickness-absence rates compared to other health professions [7]. There is also evidence that acute stress in EMT/paramedics increases medical errors [8]. It would be useful to quickly and easily identify events that are likely to have these serious sequelae in order to take measures to reduce their impact. Objective tools to identify critical incidents which are likely to result in emotional difficulties might also reduce the stigma that EMT/paramedics experience when reporting such incidents, thus facilitating timely support.

The first goal of this investigation was to develop an inventory of critical incident characteristics which are significantly associated with emotional distress at the time of an index critical incident and test the relationship of these characteristics with potential later sequelae, including slower recovery from symptoms of acute stress, and emotional symptoms occurring long after the incident. The second goal was to make this inventory as brief and useful as possible by eliminating items which did not add substantially to the strength of the associations which were found.

We chose to study both recovery from acute stress symptoms soon after a critical incident as well as occurrence of later emotional symptoms, because a chain of events may follow a critical incident. First, events which are appraised to be a greater threat than one has the resources to handle effectively elicit distress through an iterative process of appraisal, response and reappraisal [9]. Immediate (peritraumatic) distress may lead to the Acute Stress Reaction [10], which commonly includes physical arousal, distressing emotions, irritability, impaired sleep and social withdrawal, and usually returns to normal within hours or at most a few days. When the Acute Stress Reaction is prolonged, it predicts long-term outcomes of depression, PTSD, and burnout [11]. In the peritraumatic period, it is important to consider dissociation in addition to distress. While peritraumatic distress is a direct response to a stressful event, panicky feelings may also lead to peritraumatic dissociation, which is a strong predictor of later PTSD [12].

In this study, we identified the characteristics of incidents that cause EMT/paramedics’ immediate distress and subsequent symptoms in three ways. The first, and most impressionistic filter on identifying these characteristics was for paramedics to identify an index critical incident as “troubling. ” The second was to identify characteristics of these events that were associated with greater peritraumatic distress, using a validated measure of distress. The third was to identify the characteristics of events that were associated with peritraumatic dissociation (an additional expression of distress), and “downstream ” indicators of symptoms and impaired function: recovery from components of the Acute Stress Reaction and current symptoms of depression, PTSD and burnout.

## Methods

### Study design and population

We performed a cross-sectional survey of EMT/paramedics in a large urban emergency medical services (EMS) organization. The survey asked about two time periods. The first time period began at the time of an index critical incident chosen by the subject from his or her experience of work-related critical incidents (“calls that generated unusually strong feelings, either because of the incident itself, or how it was handled or some other reason ”), and extended until responses to the incident had subsided (or it was indicated that symptoms did not ever subside). The second period was the time of completing the survey (reporting of current symptoms). Front-line and supervisory EMT/paramedics were recruited to complete a survey while attending a mandatory continuing medical education program. EMT/paramedics who were on leave were informed of the study by mail. Participants were self-selected. The study was approved by the research ethics boards of both Mt. Sinai Hospital and Sunnybrook Health Sciences Centre.

### Survey content and administration

Participants completed their choice of a paper or web-based version of the survey when and where it was convenient, and returned the surveys either on-line or by mail. They were given several months to complete and return the surveys. They volunteered to sign consent forms and then complete and submit questionnaires. Upon completion, participants’ names were entered into a draw for monthly prizes worth up to $600*.*

## Choosing an index critical incident

Participants were asked to identify an index critical incident. In order to maximize opportunities for response, we offered a hierarchy of options for identifying an index incident. Participants were first asked to identify an incident that was “still troubling ”. Those who could not identify a still troubling incident were asked to identify an incident that “had been troubling in the past ”. Those who could not identify a single incident of this type were asked to describe “a composite of a number of critical incidents ”. Finally, those who were unable to describe a composite were asked to describe “one of your worst calls ”. We chose to ask our subjects about being “troubled ” by a “critical incident ” in order to use phrases that are part of EMT/paramedics’ workplace lexicon. We expected that some of these incidents might meet criteria of traumatic incidents, as defined by DSM-IV, however, we also expected that the term “critical incident ” might include a broader range of incidents.

For similar reasons we chose to ask about a broader range of outcomes than are sometimes included in studies of purely “traumatic ” incidents, such as burnout. This is because critical incidents, defined as stressful workplace incidents, may have an effect on the ability to approach work with interest, energy, and a feeling of purpose.

## Characteristics of critical incident

The investigators developed a list of 36 putative characteristics of critical incidents based on both a literature review [2-5], and focus groups which were held during a pilot for the present study. Participants reflected on the index critical incident and rated each of the 36 items as to “what degree it made the situation you are describing troubling. ” Responses were rated on a 4-point scale: 0, *does not apply*; 1 *somewhat*; 2 *quite a bit*; 3 *a lot*. For analysis, these ratings were collapsed into a dichotomous score: responses of 0 or 1 (not endorsed) or responses of 2 or 3 (endorsed). The content of the items is described in the Results section.

### Instruments

#### Responses at the time of the incident

##### Peritraumatic distress

The Peritraumatic Distress Inventory is 13-item inventory which probes emotional and physical responses at the time or immediately after a traumatic incident. It has previously demonstrated internal reliability and stability over time. We omitted one item (difficulty controlling bowel and bladder) that was least endorsed in the inventory development in police officers and had lower item-total correlations in a previous study [13]. The items have also been described by EMT/paramedics after critical incidents [4]. The scale is scored as the mean of all item scores, rated on a 4-point scale from 1 (“not at all true ”) to 4 (“extremely true ”). In the current sample, internal reliability (Cronbach’s alpha) was 0.73. Generally alpha >0.8 is considered excellent, 0.6-0.8 good, <0.6 poor. Peritraumatic distress scores were approximately normally distributed (mean 1.95 ± 0.48).

##### Peritraumatic dissociation

The Peritraumatic Dissociation Experience Questionnaire [14] is a commonly used 10-item questionnaire which probes dissociative responses during or immediately after a critical incident (e.g. “What was happening seemed unreal to me, like I was in a dream or watching a movie or a play ”). The scale is scored as the mean of responses, measured on a 5-point scale from 1 (“not at all true/does not apply ”) to 5 (“extremely true ”). In the current sample, Cronbach’s alpha was 0.85. Peritraumatic dissociation scores were non-parametrically distributed and skewed toward the minimum score (median = 1.5, inter-quartile range 1.2 – 2.1).

#### Recovery from symptoms of acute stress

##### Duration of reactions to critical incident

We measured five components of the Acute Stress Reaction which commonly occurs after extremely stressful incidents by self-report. The components measured were physical reactions (“like sweating, shaking, and pounding heart ”), distressing feelings (“like fear, anger, horror, guilt, shame, worry or sadness ”), disturbed sleep (“sleep disrupted by the incident ”), irritability (“irritable, mean or snappish ”) and social withdrawal (“if you withdrew or pulled back from other people ”) [10,15]. For each of the five components we asked, “If you had [this reaction], how long did it take before it/they were gone [or settled down, or got back to normal]? Participants chose one of seven options: (i) did not have this reaction; or returned to normal (ii) soon after the call (a few hours), (iii) by the next night, (iv) by the next week, (v) by the next month, (vi) within a few months, or (vii) still not normal.

#### Current symptoms

Current symptoms were measured separately for two time periods, first for the most recent block of shifts on-duty, and second for the most recent block of shifts off-duty. This was because the participants in pilot testing informed us that the two time periods were experienced differently, with on-duty periods evoking more symptoms. We report here on the responses during the on-duty periods because they were the most stressful and therefore the most salient.

##### Depressive symptoms

The Center for Epidemiologic Studies Depression Scale, short form (CES-D-10) is a 10-item scale in which responses rate the frequency of depressive phenomena over the most recent block of shifts worked on a 4 point scale from 0 (rarely or none of the time, less than one day) to 3 (all of the time, 5 – 7 days). CES-D-10 scores show concurrent validity with measures of positive affect (r = -.63) and poor health status (r = .37). The 10-item scale is highly correlated with the full 20-item scale, which has been validated against clinical diagnoses of depression [16]. The time period “your current or most recent block of shifts on duty ” was used rather than “over the last week ” because EMT/paramedics interviewed in the earlier phase of this research reported that perceived psychological distress was worse during blocks of shifts on duty than during blocks off duty. Cronbach’s alpha was 0.77. In the current sample, scores were approximately normally distributed (mean 7.4 ± 4.6).

##### Posttraumatic symptoms

The Impact of Events Scale-Revised, a widely used self-report measure of traumatic stress, is comprised of 22 items probing the intensity of distress associated with a particular event on a 5-point scale from 0 (not at all) to 4 (extremely). The scale is scored as the mean of item scores. The IES-R yields 3 subscales (avoidance, intrusion, and hyperarousal) and a total score. The three subscales have strong internal consistency and satisfactory test-retest reliability [17]. The correlation between the Mississippi Scale for Combat-Related PTSD, Civilian Version and the three subscales of the IES-R were: Intrusion, r = .53, Avoidance, r = .55, and Hyperarousal, r = .55 [18]. Cronbach’s alpha for the total scale was 0.91. In the current sample 56 participants (25%) identified the index IES-R event as the critical incident, 126 (55%) indicated some other experience and 46 (20%) did not specify an event. IES-R scores were non-parametrically distributed and skewed toward the minimum score (median 0.7, inter-quartile range 0.3 – 1.0).

##### Burnout

The 9-item emotional exhaustion subscale of the Maslach Burnout Inventory Human Services Survey shows strong reliability and validity [19]. Responses describe the frequency of phenomena over a long period (up to a year) on a seven-point scale from 1 (never) to 7 (every day). Burnout scores were approximately normally distributed (mean 21.8 ± 11.6) and Cronbach’s alpha was 0.92.

### Data analysis

#### Strategy to develop and validate the inventory

There is no gold standard method of indentifying the characteristics of a critical incident that are likely to cause emotional sequelae. Our strategy was to assemble an inclusive inventory of potential characteristics and then to reduce the number of items by retaining only those whose relationship with peritraumatic distress was above a threshold. Retained items were then sorted into logical categories. We categorized characteristics of events that might present such an overwhelming threat into three types: situational, systemic and personal.

Factor analysis was not appropriate because the instrument is an inventory of heterogeneous characteristics, not a scale. For example, there is no *a priori* reason to expect that an event involving one situational characteristic (e.g. a child was involved) would be more likely than any other event to also involve another situational characteristic (e.g. the situation was dangerous for me). For the same reason, the quantitative outcome of the inventory was not a score but a tally and measures of internal reliability were not appropriate. Two types of indices were derived: endorsement of any characteristic in a domain, and the total number of characteristics endorsed.

We then calculated the relationship between indices derived from the inventory and measures of the phenomena which are expected *a priori* to result from the distress caused by critical incidents: peritraumatic dissociation, the occurrence and delayed recovery from the Acute Stress Reaction and psychological symptoms occurring long after the event.

#### Development of the inventory based on relationship of items to peritraumatic distress

1. *Selection and classification of inventory items.*

The prevalence of endorsement of each item on the 36-item Critical Incident Inventory items was calculated. We identified items that were distressing at the time of the critical incident by comparing the mean intensity of peritraumatic distress among participants who did or did not endorse the item using one-way analysis of variance (ANOVA), estimating the effect size with the eta^2^ statistic. In order to reduce the number of items on the inventory, we removed items if the eta^2^ was < 0.015. The remaining characteristics were sorted into three logical domains (situational, systemic and personal characteristics) independently by two investigators (JH, RGM). Discrepancies were resolved by consensus.

2. *Prevalence of endorsing situational, systemic, and personal domains and the relationship of domains to peritraumatic distress*.

In order to define the importance of each of the domains (situational, systemic, and personal) to peritraumatic distress, we calculated the prevalence of *any* item being endorsed, and the number of items which were endorsed for each domain. The relationship between these variables and peritraumatic distress was calculated with bivariate analysis of variance and Spearman rank-order correlations respectively.

3. *Association of inventory domains with subsequent symptoms*.

We tested the associations of inventory domains with (i) peritraumatic dissociation, (ii) occurrence and recovery from components of the Acute Stress Reaction (distressing feelings, insomnia, social withdrawal, irritability, physical symptoms of arousal), and (iii) symptoms of depression, posttraumatic stress and burnout measured at a variable but longer time after the critical incident (i.e. at the time of the study, “current ”), using multivariate analysis of variance. We expected that characteristics of an incident that are validly associated with its critical nature would be *strongly* associated with the immediate impact of the incident (dissociation and prolonged Acute Stress Reaction) and *weakly* associated with current psychological symptoms at the time of the survey. Finally, we tested whether the number of characteristics endorsed was associated with the same post-incident variables using Spearman’s rank-order correlations.

## Results

Nine hundred and six EMT/paramedics were informed of the study. Of 635 individuals who signed consent forms, 243 (38.3%) completed questionnaires. Of these, 121 (49.8%) identified an incident that was “still troubling ”, 88 (36%) identified an incident that “had been troubling in the past ”, 4 (1.6%) reported on “a composite of a number of critical incidents ”, and 16 (6.6%) reported on “one of your worst calls ”. In this analysis, in order to understand the characteristics of particular critical incidents, we excluded the 4 subjects who reported on a composite index, 14 subjects who did not indicate the nature of the index incident, and 2 subjects who did not complete the Critical Incident Inventory. We report on the remaining 223 participants.

The characteristics of the EMT/paramedics who participated in this study are described in Table 1. The majority of participants (144, 64.6%) had experienced between one and five career critical incidents. Forty six (20.6%) had experienced more than 10. For most (168, 75.3%) the index incident was more than a year in the past. For comparison, the characteristics of the EMS service from which the participants were recruited were as follows: 76% male, mean age 37.5 years, mean years of service 11.4, level of training distributed as 52% level 1, 24% level 2, 21% level 3, 3% supervisors. Thus the sample of participants was similar to the EMS service as a whole except that female gender and more experienced and more highly trained EMT/paramedics were over-represented.

**Table 1:** Characteristics of 223 participating EMT/paramedics

		**Mean ± SD**	***N***	**%**
Gender	Male		142	63.7%
	Female		80	35.9%
	Not reported		1	0.4%
Marital status	Single		74	33.2%
	Married/common-law		134	60.1%
	Separated/divorced		14	6.3%
	Not reported		1	0.4%
Age (years)		37.4 ± 9.3		
Years of service		7.6 ± 3.3		
Level of training	Level 1 (EMT-D)*		95	42.6%
	Level 2 (EMT-I)*		40	17.9%
	Level 3 (EMT-P)*		82	36.8%
	Level 4 (Supervisor)		3	1.3%
	Not reported		3	1.3%
Have a permanent work partner			117	52.5%
Have a permanent work station			138	61.9%
Work in the downtown core			76	34.1%

*Note: * EMT * – Emergency Medical Technician, * D * – Defibrillator-trained, * I * – Intermediate, * P * – Paramedic.

### Development of the inventory based on relationship of items to peritraumatic distress

1. Selection and classification of inventory items

The prevalence of endorsement and relationship to peritraumatic distress were calculated for 36 characteristics of critical incidents (Table 2). Fourteen items with an effect size < 0.015 were excluded from further analysis. The remaining 22 characteristics were categorized as situational, related to the EMS organization (“systemic ”), or to the EMT/paramedics’ personal situation immediately preceding, or emotional response to, the incident (“personal ”). Categorization by two investigators was identical for 19 items (86%). Disagreement on the remaining 3 items (I was surprised by the call; factors beyond my control; end of shift) was resolved by consensus.

**Table 2:** Prevalence and effect of characteristics that made the index incident troubling

	Prevalence*		**Effect size†**	
	***N***	**%**	**eta**^**2**^	**sig.**
**Situational characteristics**				
Factors beyond my control.	140	62.8	.09	<.001
It showed how people can be cruel or neglectful	97	43.5	.02	.02
Dealing with the relatives was difficult.	88	39.5	.06	<.001
End of shift.	35	15.7	.02	.045
The situation was dangerous for me or another paramedic.	27	12.1	.09	<.001
I spent time with the patient and I got to know him – her	19	8.5	.02	.06
*Any situation characteristic*	*197*	*88.3*	*.09*	*<.001*
**Systemic characteristics**				
It was mismanaged at the time.	48	21.5	0.06	<.001
How the call was relayed - treated.	40	17.9	0.05	.001
The supervisor’s reaction.	36	16.1	0.04	.002
It was mismanaged after the incident.	36	16.1	0.02	.04
How the call was handled by dispatch.	27	12.1	0.05	.001
There was an investigation or complaint about it	27	12.1	0.03	.01
Reactions of peers.	26	11.7	0.03	.01
Inadequate equipment.	12	5.4	0.02	.06
*Any system characteristic*	*101*	*45.3*	*0.08*	*<.001*
**Personal characteristics**				
I was surprised by the call.	116	52.0	0.08	<.001
I felt helpless.	108	48.4	0.17	<.001
I felt overwhelmed.	76	34.1	0.22	<.001
I felt I didn’t do a good enough job.	47	21.1	0.07	<.001
Fatigue.	44	19.7	0.04	.004
There were cumulative work stressors in my life at the time.	33	14.8	0.07	<.001
I felt unappreciated.	24	10.8	0.05	.001
There were stresses in my personal life at the time.	23	10.3	0.07	<.001
*Any self characteristic*	*179*	*80.3*	*0.17*	*<.001*

The following items were removed because they had an effect size < 0.015: the outcome was bad (79%), a child was involved (54%), it was gruesome (44%), it was violent (38%), there were multiple casualties (22%), the patient reminded me of someone close to me (17%), lack of allied services, back-up, personnel, equipment, etc. (14%), the response of doctors and nurses on arrival at hospital (10%), over-involvement or under-involvement of allied services (10%), I don’t remember, it just was (10%), access issues (9%), the media exposure was a problem (7%), the patient was someone I knew (4%), equipment failure (2%).

† Difference in peritraumatic distress between EMT/paramedics who did or did not endorse this item.

2. Prevalence of endorsing situational, system and personal characteristics and their relationship to peritraumatic distress

**Figure 1:**
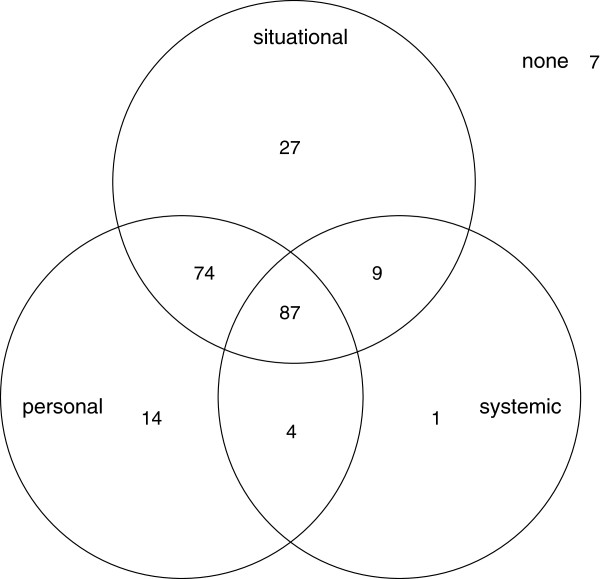
Distribution of 223 EMT/paramedics by endorsement of at least one item from each of three domains of critical incident characteristics: situational, systemic and personal characteristics.

Situational characteristics were endorsed by 197 (88.3%) participants, systemic characteristics by 101 (45.3%) and personal characteristics by 179 (80.3%). A Venn diagram (Figure 1) reveals that situations with characteristics in multiple domains were common. The combined presence of characteristics from all three domains was endorsed by 87 (39.0%) participants, while another 87 (39.0%) participants reported the presence of characteristics from two domains. The occurrence of systemic characteristics in the absence of situational or personal characteristics was reported by only one participant.

The relationship between peritraumatic distress and the three domains is presented in Table 3. Both situational and personal characteristics had significant main effects on peritraumatic distress. Neither systemic characteristics nor any of the interaction terms made a significant contribution.

**Table 3:** Relationship between endorsing any situational, systemic or personal characteristic of critical incident and peritraumatic distress

	**df**	**F**	**Sig.**
Model	7	11.9	<0.001
Situational	1	4.1	0.04
Systemic	1	0.5	0.49
Personal	1	10.3	0.002
Situational X Systemic	1	0.3	0.61
Situational X Personal	1	0.7	0.40
Systemic X Personal	1	2.2	0.14
Three-way interaction	1	0.2	0.69

The relationship between endorsing multiple characteristics and reporting peritraumatic distress was also tested. In the original 36-item inventory, there was a median of 8 characteristics endorsed to describe the index incident. In the 22-item inventory there was a median of 4 endorsed items. Peritraumatic distress was significantly associated with the number of characteristics endorsed in each domain (all p<.001). The strength of the correlation was moderate to strong for situational (rho = .52) and personal characteristics (rho = .61) as well as for the total scale in both 36-item (rho = .58) and 22-item (rho = .62) versions, but was weaker for systemic characteristics (rho = .30).

3. Relationship between inventory and other measures of the (rho=.30). 3 psychological impact of critical incidents

The relationship between endorsing any situational, personal or systemic characteristic with post-CI variables is provided in Table 4. Since interactions between the personal and situational domains were not significant in predicting peritraumatic distress, for simplicity only main effects were considered in the remainder of the analyses. The results (Table 4) show the strongest relationships are with peritraumatic dissociation, current posttraumatic symptoms, and prolonged recovery from post-critical incident distressing feelings and irritability. Weaker but significant relationships are found with prolonged recovery from post-critical incident physical arousal, social withdrawal and insomnia. The relationships between critical incident characteristics and current symptoms of depression and burnout are non-significant, although the relationship of these domains to current PTSD symptoms is both strong and significant.

**Table 4:** Relationship between any situational, systemic or personal characteristic of index incident and post- incident variables

	**Situational**		**Systemic**		**Personal**		**Model**	
	**F, df 1**	**Sig.**	**F, df 1**	**Sig.**	**F, df 1**	**Sig.**	**F, df 3**	**Sig.**
Peritraumatic dissociation	3.5	0.06	3.7	0.06	13.3	<0.001	9.2	<0.001
Post-CI prolonged physical arousal	0.0	0.95	2.0	0.16	7.5	0.007	4.0	0.009
Post-CI prolonged distressing feelings	1.3	0.26	0.4	0.54	18.4	<0.001	7.9	<0.001
Post-CI prolonged social withdrawal	3.8	0.054	2.2	0.14	3.8	0.053	4.5	0.005
Post-CI prolonged insomnia	2.1	0.15	0.3	0.56	5.7	0.02	3.4	0.02
Post-CI prolonged irritability	1.5	0.22	6.1	0.02	3.5	0.06	5.2	0.002
Current depressive symptoms	0.0	0.93	0.7	0.40	4.7	0.03	2.2	0.09
Current posttraumatic symptoms	9.6	0.002	0.1	0.73	12.6	0.001	8.8	<0.001
Current burnout symptoms	0.8	0.37	1.6	0.21	3.1	0.08	2.5	0.06

Next we tested whether the number of characteristics endorsed was associated with post-incident variables. Since systemic characteristics were more weakly related to peritraumatic distress than situational or personal characteristics and rarely occurred in the absence of situational or personal characteristics (Figure 1, n = 1, 0.5%), we compared not only the three specific domains of critical incident characteristics, but also three versions of the total inventory: the 36 original items, the 22 items which survived elimination based on effect size with respect to peritraumatic distress, and the 14 items comprising the situational and personal domains.

**Figure 2:**
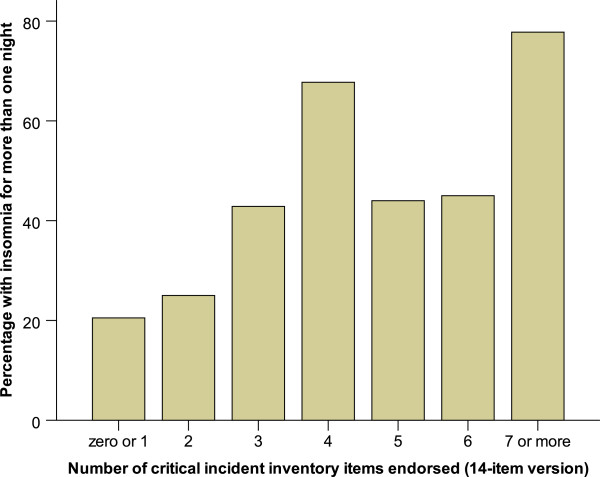
Relationship between multiplicity of endorsed items on Critical Incident Inventory (14-item version) and insomnia lasting more than one night after a critical incident.

Multiplicity of endorsed characteristics was related to both the presence of peritraumatic dissociation and prolonged recovery from the Acute Stress Reaction. The relationship between the number of characteristics endorsed and post-critical incident symptoms is presented in Table 5. In general, there is a trend to decreasing strength of correlation from (a) peritraumatic to (b) early post-incident acute distress to (c) current depressive and burnout symptoms. Endorsement of multiple personal characteristics was more strongly related to all critical incident outcomes (peritraumatic, prolonged acute distress, and current symptoms) than multiplicity of endorsement of items in the situational or systemic domains. The relationship between multiplicity of symptoms and acute post-critical incident distress is exemplified with respect to insomnia in Figure 2. With respect to current symptoms, current posttraumatic symptoms were moderately strongly related to critical incident characteristics in the situational and personal domains (Table 5). Comparing 14-item (situational + personal), 22-item (situational + personal + systemic) and 36-item (all original items) versions of the total scale indicates that the strength of relationship of critical incident characteristics and post-critical incident variables is not reduced by using the 14-item inventory.

**Table 5:** Spearman rank-order correlation between number of critical incident characteristics endorsed and post-critical incident variables

	**Situational (6-item)**	**Systemic (8-item)**	**Personal (8-item)**	**Total (36-item)**	**Total (22-item)**	**Total (14-item)**
Peritraumatic dissociation	0.35***	0.22**	0.47***	0.51***	0.46***	0.50***
Post-CI prolonged physical arousal	0.19**	0.22**	0.39***	0.37***	0.38***	0.37***
Post-CI prolonged distressing feelings	0.16*	0.16*	0.36***	0.32***	0.33***	0.32***
Post-CI prolonged social withdrawal	0.23**	0.18**	0.32***	0.35***	0.35***	0.32***
Post-CI prolonged insomnia	0.19**	0.18**	0.39***	0.41***	0.37***	0.37***
Post-CI prolonged irritability	0.23**	0.27***	0.33***	0.37***	0.35***	0.36***
Current depressive symptoms	0.11	0.21**	0.30***	0.30***	0.26***	0.29**
Current posttraumatic symptoms	0.22**	0.21**	0.39***	0.38***	0.39***	0.39***
Current burnout symptoms	0.17*	0.20**	0.24**	0.27***	0.25**	0.26**

* p<0.05; ** p<0.01; *** p<0.001.

## Discussion

The study supports the value of a 14-item inventory consisting of 6 situational and 8 personal characteristics of critical incidents, which were selected because of their association with peritraumatic distress (Table 6). Endorsement of inventory items is moderately strongly associated with peritraumatic dissociation, and more weakly associated with prolonged recovery from post-incident acute stress symptoms, and subsequent posttraumatic and depressive symptoms and burnout. This inventory is valuable for a number of reasons.

**Table 6:** The Critical Incident Inventory


**Did any of the following characteristics make the recent incident troubling for you? Please check all that apply.**	***Does not apply***	***Somewhat***	***Quite a bit***	***A lot***
Factors beyond my control.				
It showed how people can be cruel or neglectful				
Dealing with the relatives was difficult.				
End of shift.				
The situation was dangerous for me or another paramedic.				
I spent time with the patient and I got to know him or her				
I was surprised by the call.				
I felt helpless.				
I felt overwhelmed.				
I felt I didn’t do a good enough job.				
Fatigue.				
There were cumulative work stressors in my life at the time.				
I felt unappreciated.				
There were stresses in my personal life at the time.				

Firstly, it validates the importance of the EMT/paramedic’s individual experience of the incident: state of mind before the incident (e.g. feeling stressed or fatigued), appraisal of an incident (e.g. that the event is beyond his/her control), and personal internal experience of the incident (e.g. feeling helpless), as useful predictors of the acute and long-term response to the incident.

A second contribution of this 14-item inventory was testing some long-held beliefs about critical incidents. The expectation among EMT/paramedics that incidents involving a child are highly distressing [2,4] was not upheld in the development of this inventory. Specifically, although in this study the involvement of a child was believed to be at least one of the distressing characteristics in 54% of critical incidents, involvement of a child was associated with very little peritraumatic distress (effects size < 0.015). One possible explanation is that, ironically, the very fact that EMT/paramedics expect incidents involving children to be stressful renders these events less distressing, perhaps by reducing surprise. This may speak to the power of knowledge in mitigating distress. Another unexpected finding is that while systemic characteristics of critical incidents have been described in the literature, including dispatch errors [5] and lack of acknowledgement by a superior [20,21], the results of this analysis suggest that systemic characteristics do not help in identifying events as critical incidents and contribute much less to the consequences of these events than situational and personal characteristics. Thus, overall, our results may help to dispel some long-held beliefs about the nature of some characteristics of critical incidents and to emphasize the importance of others, such as personal factors.

Thirdly, the inventory offers a clear framework for identifying and reporting emotions at the time of a critical incident, which might offer EMT/paramedics and their employers an entry point into identifying and discussing a critical incident shortly after its occurrence. Optimally, the availability of valid and objective tools to identify critical incidents will result in organizational support being offered and accepted. If the Critical Incident Inventory is used by EMS organizations, it could also help to de-stigmatize the expression of vulnerable emotions after a critical incident [20]. That is, if emotions become routinely reportable items within the framework of a clear non-judgmental organizational inventory, this may help to decrease the shame surrounding their expression, which is at the heart of the culture of stigma.

It is worth noting that the inventory is more strongly associated with symptoms of posttraumatic stress than symptoms of depression and burnout measured long after the incident (Table 5). This suggests some degree of specificity for trauma-related symptoms.

With regard to the inventory itself, two further points require highlighting. One is that with the exception of the correlation to posttraumatic symptoms, the association of the inventory to other post-critical incident variables follows the hypothesized trend of stronger relationships to immediate and acute post-incident variables and weaker relationships to later symptoms. The second is that most of the index critical incidents were associated with several characteristics in the 14-item version of the Critical Incident Inventory (median = 4). The number of characteristics endorsed was strongly related to peritraumatic variables and moderately related to the duration of recovery from the Acute Stress Reaction and posttraumatic symptoms (e.g. Figure 2). This suggests that the troublesome characteristics of critical incidents can be considered to be additive contributors to a spectrum of subsequent stress syndromes and symptoms rather than simply indicators that an event “counts ” as a critical incident.

### Limitations

Confidence in the results of this study is limited by its methodology. In particular, a retrospective study is subject to recall bias of the index critical incident and post-incident variables, especially for participants for whom the index incident is further in the past. Low participation rate, self-selection of participants, and the single EMS organization surveyed, contribute uncertainty as to whether the study population is representative of all EMT/paramedics. Further research is required to replicate and expand upon these findings, particularly validation of the inventory in a different cohort than that in which it was derived.

## Conclusions

Emotional sequelae after critical incidents are associated most strongly with EMT/paramedics’ personal experience, and least with systemic characteristics. A14-item inventory identifies critical incident characteristics associated with emotional sequelae. Identifying such associations may help EMS organizations in supporting affected individuals early on and potentially mitigating the negative effects of these sequelae.

## Competing interests

The authors declare that they have no competing interests.

## Authors’ contributions

Dr. JH conceived of the study and, as principal investigator, was involved in the design, and coordinated the study. She was involved with collection of data and interpretation of data. She also wrote the manuscript. Dr. RGM was involved in the conception of the study, its design, data analysis and interpretation. He was also involved in drafting the manuscript. Dr. BS was involved in the conception of the study, its design, and acquisition of data. Dr. G was involved with the conception and design of the study. All authors read, reviewed the manuscript critically for intellectual content, and approved of the final manuscript.

## Pre-publication history

The pre-publication history for this paper can be accessed here:

http://www.biomedcentral.com/1471-227X/12/10/prepub
